# Molecular and clinical profiles of T2DM, dyslipidemia, and periodontitis: insights into inflammatory and metabolic dysregulation

**DOI:** 10.3389/fendo.2025.1574392

**Published:** 2025-05-19

**Authors:** Peace Ngozi Okoro, George Oche Ambrose, Victor Emmanuel Obaro, Chidiebere Valentine Ugwueze, J. K. Olarinoye, Olalekan A. Agede

**Affiliations:** ^1^ Department of Medicine, David Umahi Federal University Teaching Hospital, Uburu, Nigeria; ^2^ Department of Pharmacology and Therapeutics, David Umahi Federal University of Health Sciences, Uburu, Ebonyi State, Nigeria; ^3^ Department of Molecular Biology, Centre for Malaria and Other Tropical Diseases Care, University of Ilorin, Ilorin, Nigeria; ^4^ Department of Kinesiology, Nutrition, and Dietetics College of Natural and Health Sciences, University of Northern Colorado, School of Sport and Exercise Science, Greeley, CO, United States; ^5^ Department of Medicine, Alex-Ekwueme Federal University Teaching Hospital, Abakaliki, Ebonyi State, Nigeria; ^6^ Department of Medicine, Alex-Ekwueme Federal University Teaching Hospital Abakaliki, Ebonyi State & Alex-Ekwueme Federal University Ndufu Alike, Ikwo, Ebonyi, Nigeria; ^7^ Endocrinology Unit, Department of Medicine, University of Ilorin & University of Ilorin Teaching Hospital, Ilorin, Nigeria; ^8^ Department of Pharmacolgy and Therapeutics University of Ilorin, Ilorin, Nigeria

**Keywords:** T2DM, dyslipidemia, periodontitis, inflammation, lipid profiles

## Abstract

**Introduction:**

Type 2 Diabetes Mellitus (T2DM), dyslipidemia, and periodontitis are interconnected conditions that exacerbate systemic inflammation and metabolic dysregulation. Understanding the molecular and clinical profiles of these comorbidities is crucial for developing targeted interventions. This study investigates the molecular and clinical profiles of individuals with T2DM, dyslipidemia, and periodontitis to identify key markers and pathways underlying disease severity and progression.

**Methods:**

Peripheral blood mononuclear cells (PBMCs) were analyzed from five patient groups: T2DM poorly controlled with dyslipidemia and periodontitis (T2DMpoorly-DLP-H), T2DM well-controlled with dyslipidemia and periodontitis (T2DMwell-DLP-H), dyslipidemia and periodontitis (DL-P), periodontitis alone (P), and healthy controls (H). Correlations between molecular and clinical markers were assessed.

**Results:**

The T2DMpoorly-DLP-H group exhibited the most extensive molecular dysregulation, including unique upregulation of *Plasminogen Activator*, Tissue *Type (PLAT)* and consistent overexpression of *Vanin-1 (VNN1)*, a key regulator of oxidative stress. HbA1c and fasting plasma glucose were highest in this group (HbA1c >12%, glucose >300 mg/dL), correlating strongly (R² = 0.88, *p* < 0.001). In contrast, the T2DMwell-DLP-H group demonstrated reduced gene dysregulation and improved glycemic control (HbA1c ~6.5%). Sex-specific differences were observed, with females exhibiting higher glycemic markers (*p* = 0.014) and males showing elevated lipid levels (*p* = 0.021).

**Discussion:**

This study identifies *Vanin-1 (VNN1)* as a potential biomarker for systemic inflammation and highlights the role of *Plasminogen Activator, Tissue Type (PLAT)* in vascular dysfunction, emphasizing the critical importance of glycemic control in mitigating molecular and clinical dysregulation. These findings underscore the need for personalized, sex-specific strategies to manage these comorbidities effectively.

## 1 Introduction

Type 2 Diabetes Mellitus (T2DM), dyslipidemia, and periodontitis are chronic conditions that frequently coexist and share overlapping inflammatory and metabolic pathways. T2DM is characterized by hyperglycemia, insulin resistance, and systemic inflammation, leading to complications such as cardiovascular disease and nephropathy (1, 2). Dyslipidemia, marked by elevated triglycerides and cholesterol levels, is a major cardiovascular risk factor and is often exacerbated in patients with T2DM due to insulin resistance and altered lipid metabolism (2). Periodontitis, a chronic inflammatory condition of the gums, is independently associated with systemic inflammation and has been linked to worsening glycemic control in T2DM (3). Together, these conditions create a synergistic environment for metabolic and vascular dysregulation.

Evidence suggests that poor glycemic control in T2DM exacerbates systemic inflammation through the activation of pro-inflammatory pathways and the production of advanced glycation end-products (AGEs), which amplify oxidative stress and vascular damage (4). Dyslipidemia further contributes to this cascade by promoting endothelial dysfunction and increasing the risk of atherosclerosis (5). Periodontitis has been shown to induce systemic inflammation via the release of inflammatory mediators, such as interleukin-6 (IL-6) and tumor necrosis factor-alpha (TNF-α), which exacerbate insulin resistance and glycemic dysregulation (6).

Despite the well-established links between these conditions, the molecular mechanisms underlying their coexistence remain poorly understood. Identifying shared molecular markers and pathways is critical for developing targeted interventions. Previous studies have implicated genes such as *Vanin-1 (VNN1)* and *Plasminogen Activator, Tissue Type (PLAT)* in oxidative stress, inflammation, and vascular dysfunction (7, 8). Unlike classical pro-inflammatory cytokines such as TNF-α, IL-1β, and IL-6, which reflect acute immune activation, VNN1 and PLAT represent upstream mediators of oxidative and vascular stress (9). VNN1 is involved in pantetheinase activity and modulates glutathione levels, contributing to redox balance and chronic inflammatory signaling (10). PLAT, a serine protease, plays a key role in fibrinolysis and endothelial repair, making both genes valuable for capturing the molecular crosstalk between metabolic, inflammatory, and vascular pathways in T2DM, dyslipidemia, and periodontitis (11). However, integrated analyses combining molecular and clinical data to elucidate these relationships are limited.

This study aims to investigate the molecular and clinical profiles of patients with T2DM, dyslipidemia, and periodontitis to identify key pathways and biomarkers underlying disease severity. The findings provide insights into the interplay between inflammation, oxidative stress, and metabolic dysregulation, highlighting potential targets for personalized interventions.

## 2 Methodology

### 2.1 Study design and dataset

This study employed a bioinformatics approach to investigate the molecular profiles of individuals affected by Type 2 Diabetes Mellitus (T2DM), dyslipidemia, and periodontitis. The dataset used for the analysis was retrieved from the Gene Expression Omnibus (GEO) database with accession number GSE156993. This dataset includes microarray-based gene expression profiles of peripheral blood mononuclear cells (PBMCs) collected from five groups: T2DM poorly controlled with dyslipidemia and periodontitis (T2DMpoorly-DLP-H), T2DM well-controlled with dyslipidemia and periodontitis (T2DMwell-DLP-H), dyslipidemia and periodontitis (DL-P), periodontitis only (P), and healthy controls (H).

### 2.2 Data processing and normalization

The raw microarray data were downloaded and preprocessed in R programming language using the Bioconductor packages. The affy and oligo packages (12, 13) were used for background correction, normalization, and summarization of the probe-level data. Gene expression was normalized using the Robust Multi-Array Average (RMA) algorithm to ensure comparability across samples. Quality control metrics, including boxplots and principal component analysis (PCA), were generated to assess sample consistency and eliminate outliers.

### 2.3 Differential gene expression analysis

Differential expression analysis was performed using the limma package (14). Pairwise comparisons were conducted to identify differentially expressed genes (DEGs) between each patient group and healthy controls. Genes with an adjusted p-value < 0.05 and |log2 fold change| > 1 were considered significantly differentially expressed. Volcano plots and Box plots were generated using the ggplot2 package (15), to visualize the distribution and clustering of DEGs.

### 2.4 Venn diagram analysis

Shared and unique DEGs across groups were identified and visualized using Venn diagrams, generated with the VennDiagram package (16). This analysis highlighted common molecular mechanisms and group-specific pathways underlying the comorbidities.

### 2.5 Correlation and statistical analysis

Correlation analysis was performed between clinical parameters, such as fasting plasma glucose, HbA1c, triglycerides, and total cholesterol, using the corrplot package (17). Pearson correlation coefficients and heatmaps were used to assess relationships among variables. ANOVA followed by Tukey’s HSD *post-hoc* tests was conducted using the stats package to compare HbA1c levels among groups.

### 2.6 Gene prioritization and biomarker identification

Key genes, such as VNN1, were prioritized based on their consistent expression across groups and functional relevance to oxidative stress and inflammation. Functional annotations and pathway involvement of prioritized genes were further validated using the AnnotationDbi package (18).

### 2.7 Software and tools

All analyses were conducted in R version 4.3.0. Visualization and statistical analyses were performed using the tidyverse (19) and Bioconductor ecosystems to ensure reproducibility and comprehensive reporting of results.

### 2.8 Ethical considerations

As the study involved publicly available data, no ethical approval was required. The dataset (GSE156993) adhered to ethical guidelines for data sharing and use.

## 3 Results

### 3.1 Glycemic control across study groups

**Figure 1:**
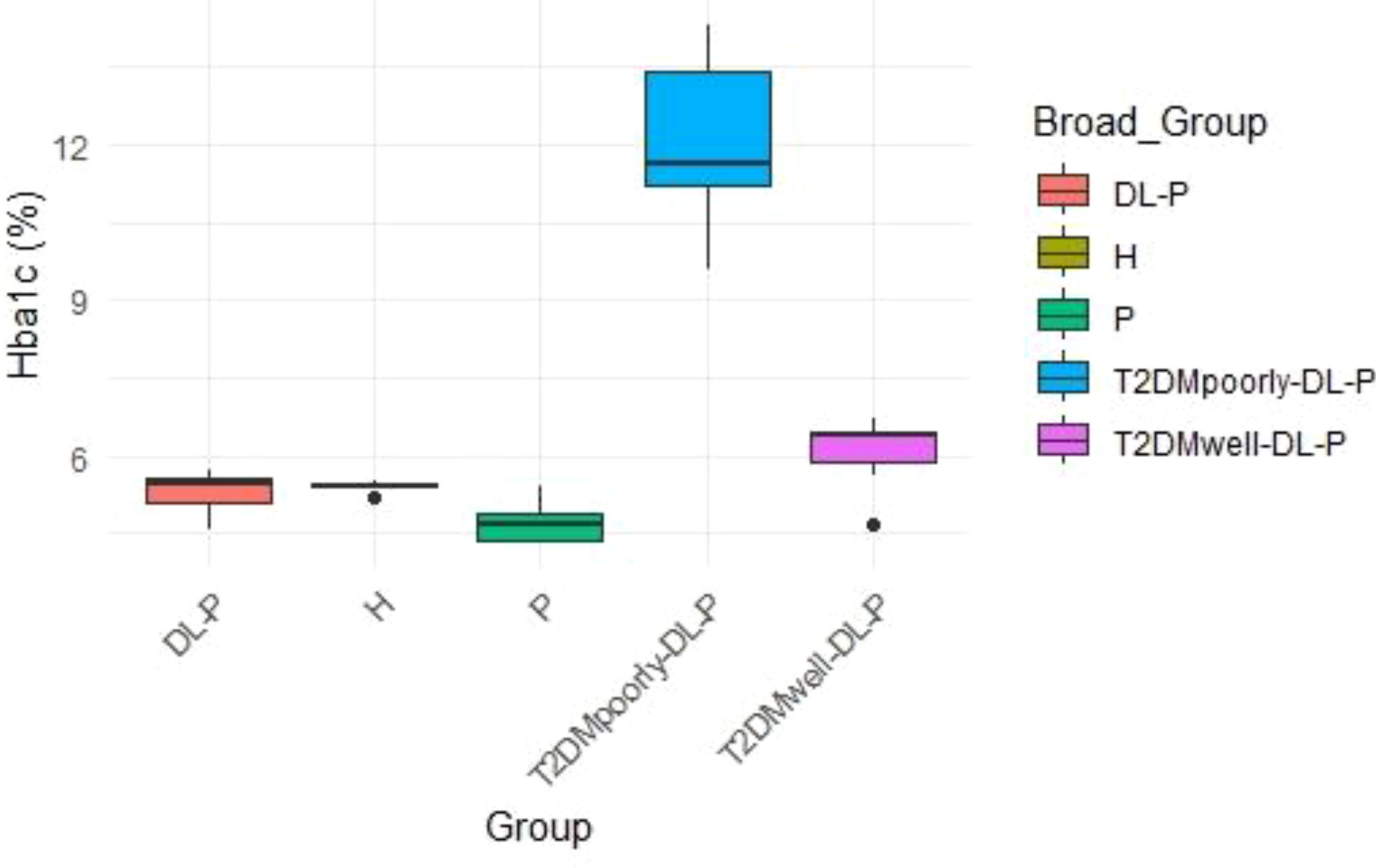
The distribution of HbA1c levels across the groups.

The distribution of HbA1c levels across the groups is presented in **Figure 1**. The ‘T2DMpoorly-DL-P’ group exhibited significantly higher HbA1c levels (median > 12%) compared to all other groups, indicating poor glycemic control. In contrast, the ‘T2DMwell-DL-P’ group had HbA1c levels clustered around 6.5%, reflecting effective glycemic management. The non-diabetic groups, including ‘DL-P’, ‘H’, and ‘P’, demonstrated relatively lower and consistent HbA1c levels, with medians below 6.0%. This trend suggests that these groups either represent individuals with healthy glycemic profiles or those with well-managed conditions.

### 3.2 Fasting plasma glucose across study groups

**Figure 2:**
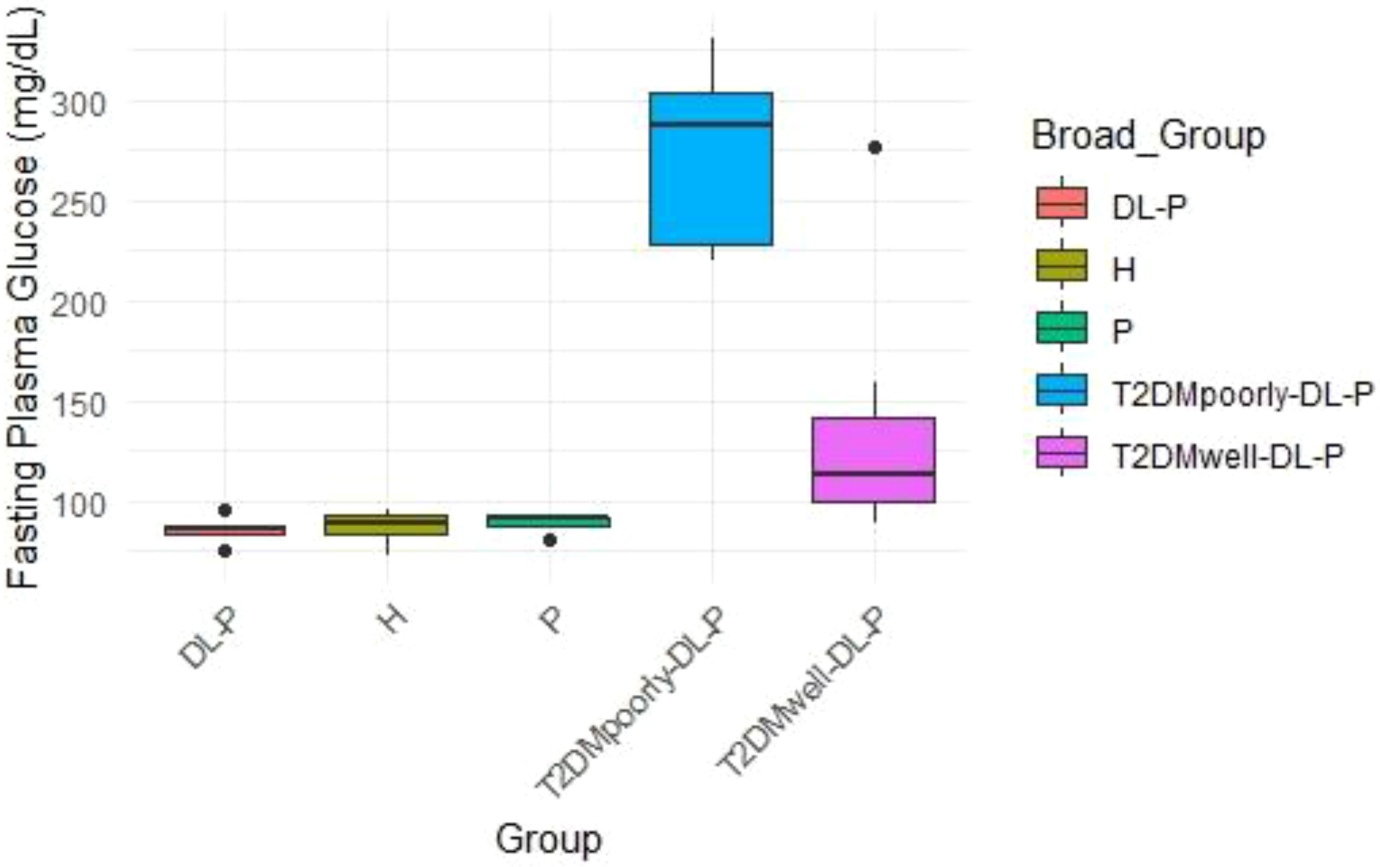
Boxplot analysis of fasting plasma glucose across all the group.

Boxplot analysis of fasting plasma glucose (**Figure 2**) revealed the highest values in the ‘T2DMpoorly-DL-P’ group, with levels exceeding 300 mg/dL in some individuals. These values are indicative of poorly managed diabetes. Conversely, the ‘T2DMwell-DL-P’ group showed moderate fasting glucose levels within the range of 90–150 mg/dL, consistent with improved glycemic control. The ‘DL-P’, ‘H’, and ‘P’ groups demonstrated fasting glucose levels below 100 mg/dL, characteristic of non-diabetic or well-controlled individuals.

### 3.3 Correlation between fasting plasma glucose and HbA1c

**Figure 3:**
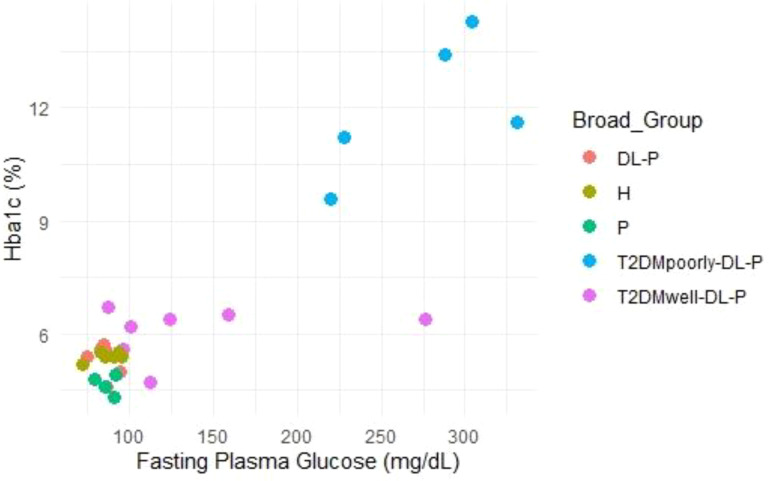
Relationship between fasting plasma glucose and HbA1c.

The scatter plot (**Figure 3**) illustrates a strong positive correlation between fasting plasma glucose and HbA1c levels (R² = 0.88). Participants in the ‘T2DMpoorly-DL-P’ group displayed higher fasting glucose levels, which corresponded to elevated HbA1c levels, further emphasizing poor glycemic control. The ‘T2DMwell-DL-P’ group clustered around lower HbA1c values (<7%) and fasting glucose levels (<150 mg/dL), indicative of effective glycemic management. Non-diabetic groups (‘DL-P’, ‘H’, and ‘P’) consistently showed low values for both parameters, affirming their healthy or controlled states.

### 3.4 HbA1c distribution by group

**Figure 4:**
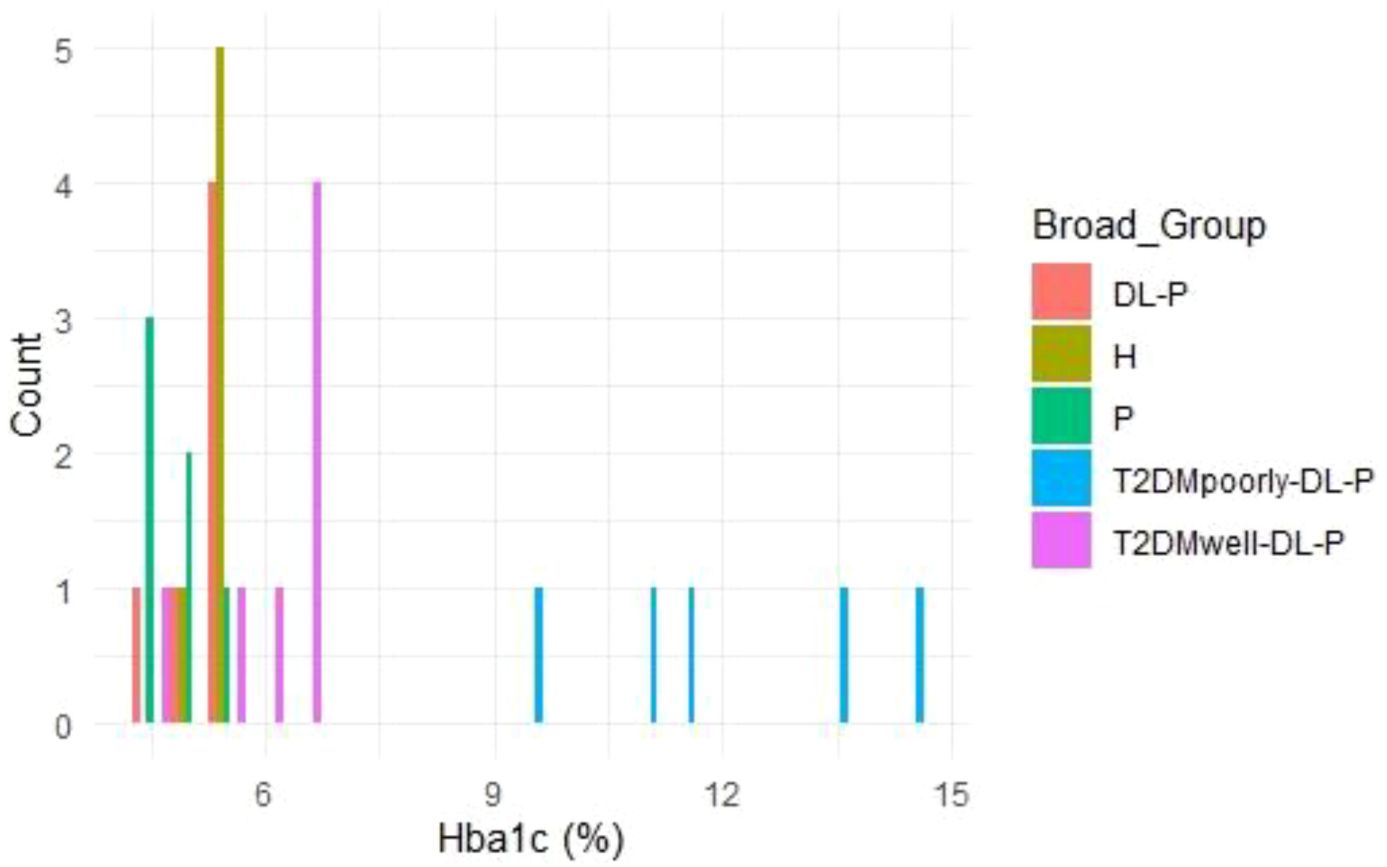
The frequency distribution of HbA1c levels across study groups.

The frequency distribution of HbA1c levels across study groups is depicted in **Figure 4**. The majority of individuals in the ‘T2DMpoorly-DL-P’ group demonstrated HbA1c levels >12%, reflecting severe hyperglycemia. In contrast, the ‘T2DMwell-DL-P’ group primarily exhibited HbA1c levels around 6.5%, suggesting that glycemic control measures were effective for most participants. For the ‘DL-P’, ‘H’, and ‘P’ groups, HbA1c levels were predominantly below 6%, consistent with non-diabetic or well-controlled profiles.

### 3.5 Correlation analysis

The correlation results reveals the interrelationships between fasting plasma glucose, HbA1c, total cholesterol, and triglycerides. HbA1c was highly correlated with fasting plasma glucose (r = 0.88), underscoring the strong association between these parameters. Additionally, triglycerides demonstrated a moderate positive correlation with total cholesterol (r = 0.85), suggesting dyslipidemia in certain groups. The lack of significant correlations between lipid parameters and HbA1c or fasting plasma glucose suggests that hyperglycemia and dyslipidemia may be independently regulated.

An ANOVA test was conducted to compare Hba1c levels among five groups: `T2DMpoorly-DL-P`, `T2DMwell-DL-P`, `DL-P`, `P`, and `H`. Results revealed a statistically significant difference among the groups, F (4, 25) = 63.62, p < 0.001. Tukey’s HSD *post-hoc* test identified the following pairwise comparisons as significant (**Table 1**).

**Table 1:** Group comparisons of Hba1c levels.

Comparison	Mean difference	95% CI	P-value
T2DMpoorly-DL-P vs. DL-P	6.72	[5.19, 8.25]	< 0.001
T2DMpoorly-DL-P vs. H	6.62	[5.09, 8.15]	< 0.001
T2DMpoorly-DL-P vs. P	7.30	[5.77, 8.83]	< 0.001
T2DMpoorly-DL-P vs. T2DMwell-DL-P	-5.95	[-7.43, -4.47]	< 0.001

A Pearson correlation matrix revealed strong associations between key clinical variables (**Table 2**).

**Table 2:** Correlation analysis.

Variable 1	Variable 2	Correlation coefficient
Fasting Plasma Glucose	Hba1c	0.88
Total Cholesterol	Triglycerides	0.85
Fasting Plasma Glucose	Total Cholesterol	0.49
Hba1c	Total Cholesterol	0.46

Clinical characteristics were stratified by sex. Female participants exhibited higher fasting plasma glucose and Hba1c levels compared to males, whereas males showed higher triglycerides and total cholesterol levels (**Table 3**).

**Table 3:** Sex-specific analysis.

Sex	Fasting plasma glucose (mg/dL)	Hba1c (%)	Total cholesterol (mg/dL)	Triglycerides (mg/dL)
Female	139.00	7.02	223.00	167.00
Male	118.00	5.82	234.00	226.00

### 3.6 Differential gene expression analysis

The total number of genes expressed across all groups was 54,675 (**Supplementary Data Sheet S1**). Differential gene expression analysis revealed significant gene expression changes (|log2 fold change| > 1, adjusted p-value < 0.05) in all groups when compared to healthy controls. A total of 1,610 genes were significantly expressed in the Dyslipidemia + Periodontitis (DLP-H) group (**Supplementary Data Sheet S2**). In the Periodontitis (P-H) group, 1,682 genes were identified as significantly expressed (**Supplementary Data Sheet S3**). The T2DM Poorly Controlled + Dyslipidemia + Periodontitis (T2DMpoorly-DLP-H) group exhibited the highest number of significant genes, with 3,117 differentially expressed (**Supplementary Data Sheet S4**). In contrast, the T2DM Well-Controlled + Dyslipidemia + Periodontitis (T2DMwell-DLP-H) group showed 2,179 (**Supplementary Data Sheet S5**) significantly expressed genes, which was fewer than the poorly controlled counterpart but still substantial.

**Figure 5:**
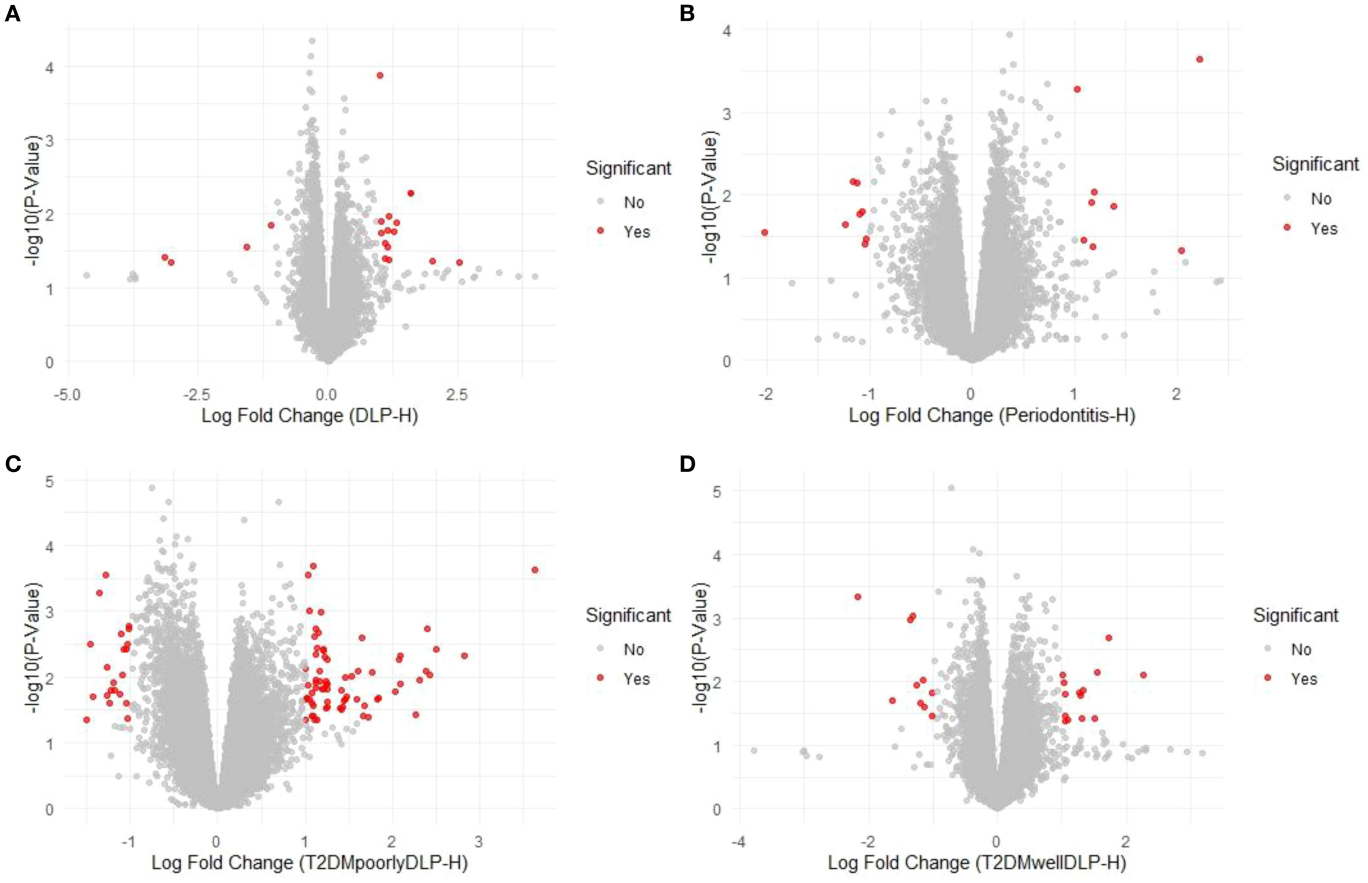
Volcano plots comparing gene expression profiles in each group relative to healthy controls. **(A)** DLP-H, **(B)** P-H, **(C)** T2DMpoorly-DLP-H, and **(D)** T2DMwell-DLP-H.

Volcano plots were generated to visualize the distribution of differentially expressed genes in each group relative to healthy controls (**Figures 5A–D**). These plots illustrate the relationship between log2 fold change and -log10 p-value, highlighting the significant gene expression changes. The DLP-H group demonstrated significant upregulation of genes primarily associated with lipid metabolism and immune activation pathways, suggesting a strong interplay between metabolic and inflammatory responses. In the P-H group, significant genes were predominantly linked to localized inflammatory responses characteristic of periodontitis.

**Figure 6:**
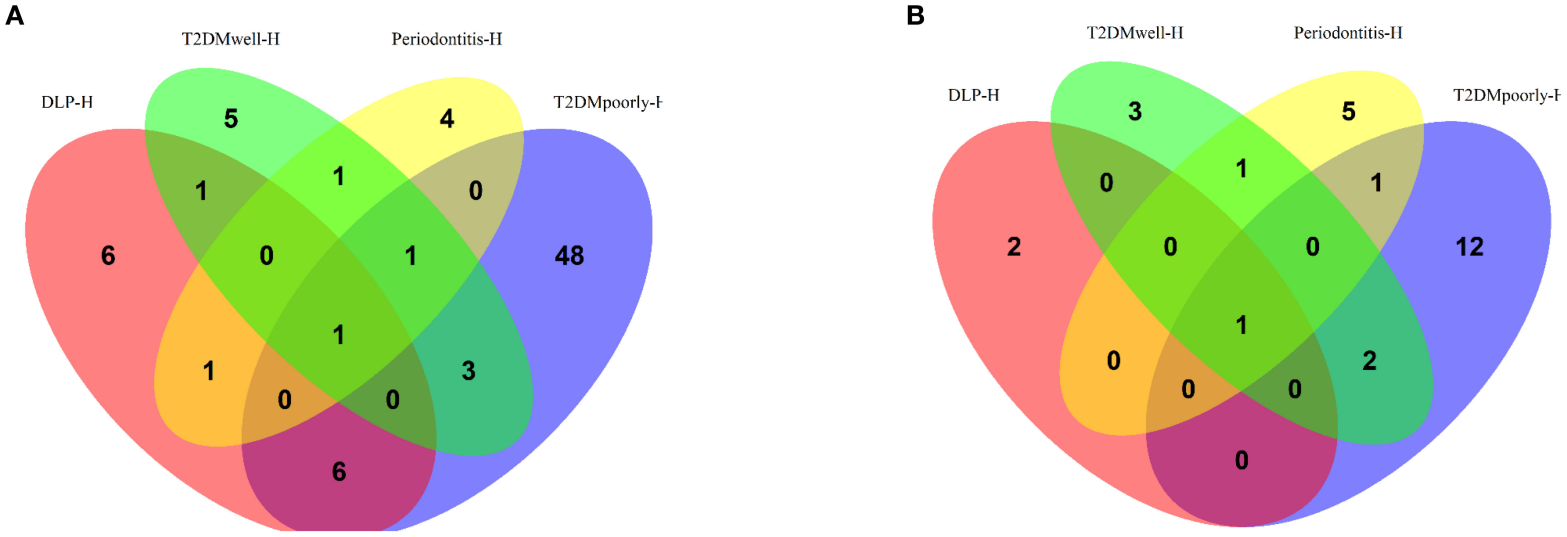
**(A)** Venn diagram for upregulated genes across DLP-H, Periodontitis-H, T2DMwell-DLP-H, and T2DMpoorly-DLP-H. **(B)** Venn diagram for downregulated genes across the same groups.

To identify shared and unique upregulated genes among the groups, a Venn diagram analysis was performed (**Figure 6A**). The T2DMpoorly-DLP-H group exhibited the highest number of uniquely upregulated genes (48 genes), reflecting the severe metabolic and inflammatory disturbances associated with poor glycemic control. The Periodontitis-H group displayed 4 uniquely upregulated genes, indicative of localized inflammation. In contrast, the T2DMwell-DLP-H group had 5 unique upregulated genes, showing a distinct profile compared to the poorly controlled group. The DLP-H group had 6 unique upregulated genes, likely associated with the combined effects of dyslipidemia and periodontitis. Importantly, one gene, VNN1 (Vanin-1), was consistently overexpressed across all groups, with log2 fold changes of 1.59, 1.53, 1.33, and 1.38 in DLP-H, T2DMpoorly-DLP-H, T2DMwell-DLP-H, and Periodontitis-H, respectively. This gene reached statistical significance in all comparisons (p-values: 0.0052, 0.0096, 0.0137, and 0.0136, respectively) and had a consistent effect size, suggesting its central role in inflammatory and immune-related pathways.

To identify consistently downregulated genes across all groups, a Venn diagram analysis was performed (**Figure 6B**). Two genes were consistently downregulated across all groups, both achieving statistical significance. The first gene, with an unknown ID, exhibited log2 fold changes of -1.10, -1.17, -1.32, and -1.09 in the DLP-H, T2DMpoorly-DLP-H, T2DMwell-DLP-H, and Periodontitis-H groups, respectively. This gene had p-values of 0.0157, 0.0152, 0.0174, and 0.0168, respectively, and was associated with pathways involved in inflammatory suppression and metabolic regulation.

The second gene, with an unknown ID too, showed log2 fold changes of -1.57, -1.50, -0.70, and 0.07 across the same groups. Despite these variations, its consistent downregulation was statistically significant (p-values: 0.0287, 0.0441, 0.2911, and 0.9191).

To further investigate the differential gene expression, the top 10 most significant genes for each group compared to healthy controls were identified based on the -log10(p-value) of their expression levels.

**Figure 7:**
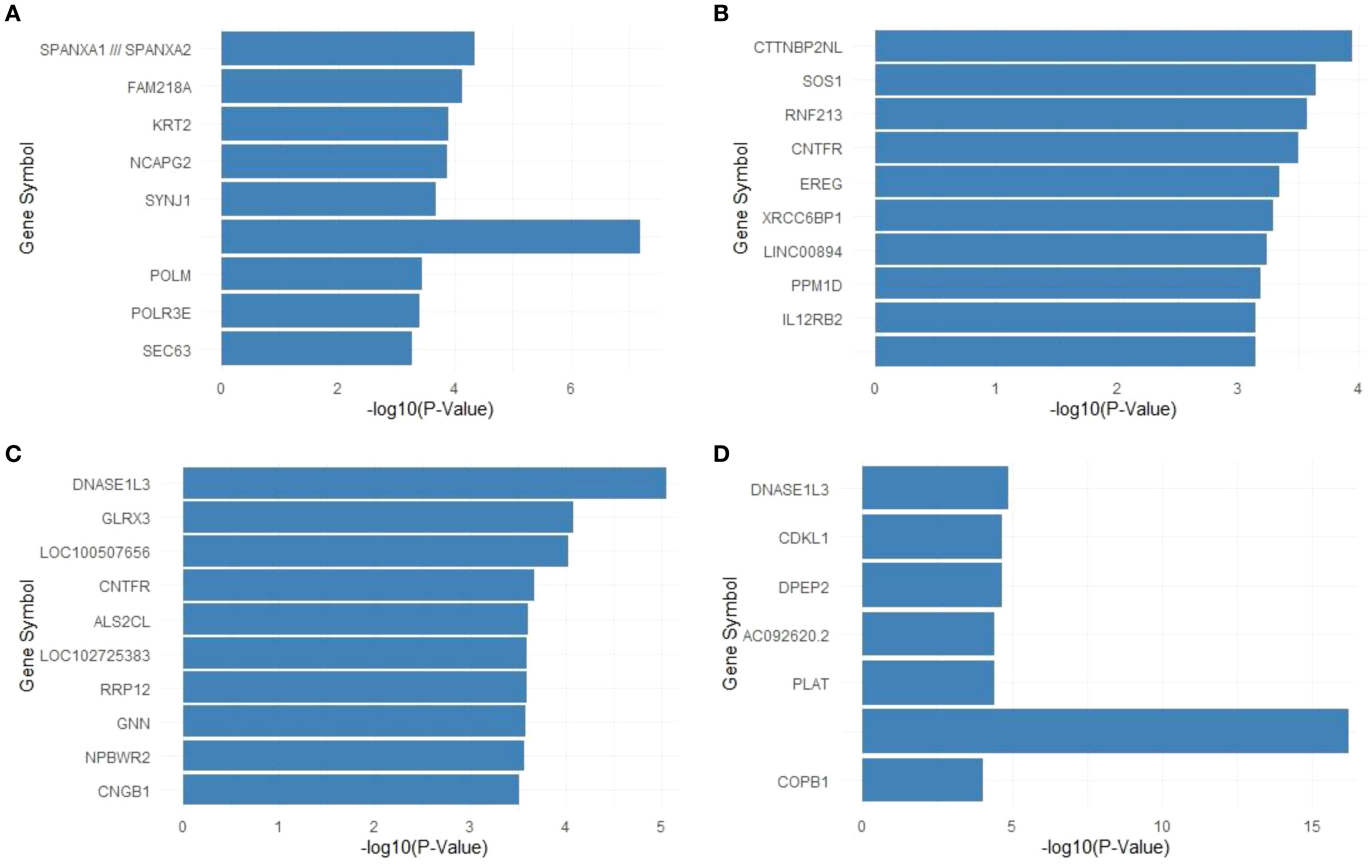
**(A)** Top 10 significant genes for DLP-H vs. Healthy. **(B)** Top 10 significant genes for Periodontitis-H vs. Healthy. **(C)** Top 10 significant genes for T2DMwell-DLP-H vs. Healthy. **(D)** Top 10 significant genes for T2DMpoorly-DLP-H vs. Healthy.

The top significant genes in the DLP-H group included SPANXA1/SPANXA2, FAM218A, KRT2, and SYNJ1, with SYNJ1 showing the highest significance level (-log10(p-value) > 6; **Figure 7A**). These genes are predominantly associated with lipid metabolism and immune regulation pathways, consistent with the dual impact of dyslipidemia and periodontitis.

In the Periodontitis group, the most significant genes included CTTNBP2NL, SOS1, RNF213, and IL12RB2 (**Figure 7B**). These genes are strongly linked to inflammatory responses and immune signaling, characteristic of localized periodontal disease.

The top significant genes in the T2DMwell-DLP-H group were dominated by genes such as DNASE1L3, GLRX3, and CNTFR, with DNASE1L3 exhibiting the highest statistical significance (-log10(p-value) > 4; **Figure 7C**). These genes reflect mechanisms related to glucose regulation and systemic inflammatory control associated with well-managed diabetes.

The T2DMpoorly-DLP-H group demonstrated the most pronounced differential gene expression, with PLAT showing an exceptionally high significance level (-log10(p-value) > 15; **Figure 7D**). Other significant genes included DNASE1L3, CDKL1, and DPEP2, highlighting the compounding effects of poor glycemic control, dyslipidemia, and periodontitis on molecular dysregulation.

## 4 Discussion

This study provides a comprehensive analysis of the molecular and clinical profiles of individuals affected by Type 2 Diabetes Mellitus (T2DM), dyslipidemia, and periodontitis, either alone or in combination. The findings reveal distinct and shared patterns of gene expression and clinical biomarkers, offering novel insights into the interplay between glycemic control, lipid metabolism, and immune response in these conditions.

### 4.1 Molecular insights into inflammatory and metabolic dysregulation

The molecular analysis revealed significant alterations in gene expression across the study groups, with the T2DMpoorly-DLP-H group demonstrating the most extensive dysregulation. This group exhibited a large number of uniquely upregulated and downregulated genes, highlighting the compounded effects of poorly managed hyperglycemia, dyslipidemia, and chronic inflammation associated with periodontitis. These findings align with earlier studies demonstrating that poorly controlled T2DM exacerbates inflammatory responses and metabolic derangements, increasing the risk of complications (20).

The consistent overexpression of VNN1 (Vanin-1) across all groups underscores its role as a central molecular marker in the studied conditions. VNN1, a pantetheinase enzyme, plays a key role in regulating oxidative stress, inflammation, and tissue repair by modulating cysteamine levels and the production of reactive oxygen species (ROS). Elevated levels of VNN1 have been linked to chronic inflammation and metabolic disorders, such as obesity, T2DM, and non-alcoholic fatty liver disease (NAFLD) (21, 22). In the context of T2DM and dyslipidemia, VNN1 upregulation likely exacerbates oxidative stress and inflammatory cascades, contributing to the systemic metabolic dysregulation observed across the groups. Previous studies have identified VNN1 as a biomarker for diabetic nephropathy and as a therapeutic target for reducing oxidative damage in metabolic disorders (23). Its consistent overexpression in this study suggests a shared pathogenic pathway among the conditions, emphasizing its potential as a therapeutic target.

In contrast to the extensive dysregulation observed in the T2DMpoorly-DLP-H group, the T2DMwell-DLP-H group demonstrated fewer gene expression changes. This finding highlights the mitigating effects of glycemic control on systemic inflammation and metabolic dysregulation. Glycemic management has been shown to reduce the expression of pro-inflammatory genes and restore metabolic homeostasis, as evidenced by reductions in markers such as interleukin-6 (IL-6) and tumor necrosis factor-alpha (TNF-α) in well-controlled diabetes (12). The observed differences between the well-controlled and poorly controlled groups underscore the critical role of glycemic control in modulating disease severity and preventing molecular damage.

Additionally, the analysis of consistently downregulated genes revealed a subset of genes involved in immune regulation and metabolic pathways. These genes, which were shared across the study groups, reflect the systemic impact of T2DM, dyslipidemia, and periodontitis on molecular homeostasis. Downregulated genes in pathways such as lipid metabolism and immune cell trafficking suggest a disruption in normal cellular functions, consistent with findings from prior research on chronic inflammation and metabolic disorders (24, 25).

Together, these findings highlight the interplay between inflammation, oxidative stress, and metabolic dysregulation in these conditions. The significant gene expression changes, particularly in the poorly controlled T2DM group, emphasize the compounded impact of hyperglycemia and dyslipidemia in driving systemic inflammation and immune dysfunction. These insights reinforce the importance of integrated disease management strategies to mitigate the molecular and clinical burden of these comorbid conditions.

### 4.2 Clinical correlates of glycemic control and dyslipidemia

The clinical data strongly corroborate the molecular findings, highlighting the critical role of glycemic control in modulating disease severity and metabolic homeostasis. The T2DMpoorly-DLP-H group exhibited markedly higher HbA1c levels (median > 12%) and fasting plasma glucose concentrations (>300 mg/dL) compared to all other groups. These findings are indicative of severe hyperglycemia and poor disease management, consistent with previous reports linking poorly controlled T2DM to heightened risks of inflammation, endothelial dysfunction, and long-term complications such as cardiovascular disease (26, 27). The strong positive correlation between HbA1c and fasting plasma glucose (R² = 0.88) further emphasizes the interdependence of these markers in assessing glycemic control and the risk of complications (28).

In contrast, the T2DMwell-DLP-H group demonstrated significantly improved glycemic control, with HbA1c levels clustered around 6.5% and fasting glucose values below 150 mg/dL. These results reflect the success of effective glycemic management strategies, such as pharmacological interventions, lifestyle modifications, or both. Achieving HbA1c levels below 7% has been associated with reduced risks of microvascular complications, as shown in landmark studies like the UKPDS and ADVANCE trials (29, 30). The non-diabetic groups, including DL-P, H, and P, exhibited consistently low HbA1c (<6%) and fasting glucose levels (<100 mg/dL), indicative of either healthy glycemic profiles or well-managed conditions. These data reaffirm the importance of early intervention and stringent glycemic targets in reducing systemic inflammation and preventing disease progression.

Interestingly, lipid parameters, including triglycerides and total cholesterol, demonstrated a moderate positive correlation (r = 0.85), consistent with dyslipidemia in certain groups. However, the lack of significant correlations between lipid levels and glycemic markers (HbA1c and fasting plasma glucose) suggests that hyperglycemia and dyslipidemia may be regulated through distinct yet overlapping pathways. This observation aligns with findings which identified independent regulatory mechanisms governing lipid metabolism and glucose homeostasis (31). Dyslipidemia is characterized by an imbalance in triglyceride-rich lipoproteins and low-density lipoproteins, often exacerbated by insulin resistance (32). However, glucose regulation is predominantly mediated by insulin sensitivity and pancreatic beta-cell function, highlighting the mechanistic divergence between these processes (6).

Further stratification of clinical data by sex revealed notable differences, with female participants exhibiting higher fasting plasma glucose and HbA1c levels compared to males. These findings are consistent with earlier studies suggesting that women with T2DM may experience greater challenges in achieving glycemic targets, potentially due to hormonal fluctuations and reduced insulin sensitivity (33, 34). Conversely, male participants showed elevated levels of triglycerides and total cholesterol, aligning with the known sex-based differences in lipid metabolism and cardiovascular risk (35). Such differences underscore the importance of personalized treatment strategies tailored to the unique physiological and hormonal contexts of male and female patients.

### 4.3 Sex-specific differences in clinical profiles

Stratification of clinical characteristics by sex revealed notable differences, providing additional insights into the interplay between metabolic dysregulation and sex-specific physiology. Female participants exhibited significantly higher fasting plasma glucose and HbA1c levels compared to males, consistent with previous findings indicating that women with diabetes may experience more pronounced difficulties in achieving glycemic targets. This discrepancy is often attributed to hormonal influences, particularly the effects of estrogen on glucose metabolism and insulin sensitivity, as well as sex-specific differences in fat distribution and inflammatory responses (36, 37). Elevated HbA1c levels in women have been associated with a higher risk of microvascular complications, including diabetic nephropathy and retinopathy, emphasizing the need for tailored glycemic management strategies in female patients (5).

In contrast, male participants exhibited higher levels of triglycerides and total cholesterol, both of which are well-established risk factors for cardiovascular disease (34, 36). These findings align with studies suggesting that men with diabetes are more likely to exhibit atherogenic dyslipidemia, characterized by elevated triglycerides and low HDL cholesterol, due to differences in lipid metabolism (38). This lipid profile predisposes men to coronary artery disease and other cardiovascular complications, underscoring the importance of lipid-lowering interventions in male patients.

The observed sex-based differences in metabolic profiles highlight the necessity of sex-specific approaches in managing diabetes and related conditions. Such approaches should account for the differential impact of hormonal regulation, body composition, and lipid metabolism on disease progression and treatment outcomes.

### 4.4 Integration of molecular and clinical findings

The integration of molecular and clinical data in this study provides a comprehensive understanding of the pathophysiological mechanisms underlying T2DM, dyslipidemia, and periodontitis, both individually and in combination. The strong correlation between clinical markers of hyperglycemia, such as HbA1c and fasting plasma glucose, and the molecular profiles observed in the T2DM groups underscores the systemic impact of poor glycemic control on inflammatory and metabolic pathways. The upregulation of genes such as PLAT (plasminogen activator) in the T2DMpoorly-DLP-H group reflects the increased fibrinolytic activity and vascular inflammation associated with poorly managed diabetes. Elevated expression of PLAT has been linked to endothelial dysfunction and a heightened risk of thrombotic events, consistent with the vascular complications frequently observed in patients with uncontrolled diabetes (23, 39).

A novel and significant finding from this study is the consistent overexpression of VNN1 (Vanin-1) across all study groups. This gene, a critical regulator of oxidative stress and inflammation, has been implicated in chronic inflammatory diseases and metabolic disorders, including diabetes and non-alcoholic fatty liver disease (40, 41). The identification of VNN1 as a shared marker across diverse patient groups suggests its potential as a unifying biomarker for inflammatory and metabolic dysregulation, regardless of the specific combination of comorbidities.

Additionally, the differential expression of genes such as SYNJ1 and PLAT highlights the role of specific molecular pathways in driving disease severity and complications. SYNJ1, implicated in vesicular trafficking and lipid metabolism, may contribute to the metabolic and inflammatory dysregulation observed in the DLP-H group. These findings suggest that targeting specific pathways, such as oxidative stress (via VNN1) or vascular inflammation (via PLAT), may provide novel therapeutic avenues for managing T2DM and its complications.

The distinct molecular and clinical profiles observed in the T2DMpoorly-DLP-H group emphasize the critical need for aggressive and multifaceted management strategies. Addressing systemic inflammation, oxidative stress, and metabolic dysregulation through a combination of glycemic control, lipid-lowering therapies, and anti-inflammatory interventions may reduce the burden of complications in high-risk populations. The findings from this study contribute to a growing body of evidence highlighting the importance of personalized medicine approaches in addressing the heterogeneity of metabolic disorders.

## 5 Conclusion

This study highlights the interplay between glycemic control, lipid metabolism, and inflammation in T2DM, dyslipidemia, and periodontitis. The consistent overexpression of VNN1 as a biomarker underscores its potential in disease management. Findings emphasize personalized, sex-specific approaches and targeted therapies addressing oxidative stress, inflammation, and metabolic dysregulation to mitigate complications.

## 6 Limitations and future directions

While this study provides valuable insights, it is limited by its cross-sectional design, which precludes causal inferences. Additionally, the sample size for certain subgroups may limit the generalizability of findings. Future longitudinal studies with larger cohorts are warranted to validate these results and explore the therapeutic potential of the identified biomarkers.

## Data Availability

The original contributions presented in the study are included in the article/**Supplementary Material**. Further inquiries can be directed to the corresponding author.
